# Paraprobiotics and Postbiotics of *Lactobacillus delbrueckii* CIDCA 133 Mitigate 5-FU-Induced Intestinal Inflammation

**DOI:** 10.3390/microorganisms10071418

**Published:** 2022-07-14

**Authors:** Viviane Lima Batista, Luís Cláudio Lima De Jesus, Laísa Macedo Tavares, Fernanda Lima Alvarenga Barroso, Lucas Jorge da Silva Fernandes, Andria dos Santos Freitas, Monique Ferrary Americo, Mariana Martins Drumond, Pamela Mancha-Agresti, Enio Ferreira, Juliana Guimarães Laguna, Luiz Carlos Júnior Alcantara, Vasco Azevedo

**Affiliations:** 1 Department of Genetics, Ecology and Evolution, Federal University of Minas Gerais, Belo Horizonte 31270-901, Brazil; vivianelimabio@gmail.com (V.L.B.); lc.luiis@yahoo.com.br (L.C.L.D.J.); macedolaisa@gmail.com (L.M.T.); fernanda_alima@hotmail.com (F.L.A.B.); lucazjorge@hotmail.com (L.J.d.S.F.); andria.sfreitas@gmail.com (A.d.S.F.); moniquefamerico@gmail.com (M.F.A.); jujulaguna@gmail.com (J.G.L.); 2 Department of Biological Sciences, Federal Center for Technological Education of Minas Gerais, Belo Horizonte 30421-169, Brazil; mmdrumond@gmail.com (M.M.D.); p.mancha.agresti@gmail.com (P.M.-A.); 3 Department of General Pathology, Federal University of Minas Gerais, Belo Horizonte 31270-901, Brazil; enioferreira@icb.ufmg.br; 4 Flavivirus Laboratory, Oswaldo Cruz Institute, Oswaldo Cruz Foundation, Rio de Janeiro 21040-900, Brazil

**Keywords:** mucositis, chemotherapy, intestinal damage, heat-killed bacteria, cell-free supernatant, immunomodulation, epithelial barrier markers

## Abstract

Intestinal mucositis is a commonly reported side effect in oncology practice. Probiotics are considered an excellent alternative therapeutic approach to this debilitating condition; however, there are safety questions regarding the viable consumption of probiotics in clinical practice due to the risks of systemic infections, especially in immune-compromised patients. The use of heat-killed or cell-free supernatants derived from probiotic strains has been evaluated to minimize these adverse effects. Thus, this work evaluated the anti-inflammatory properties of paraprobiotics (heat-killed) and postbiotics (cell-free supernatant) of the probiotic *Lactobacillus delbrueckii* CIDCA 133 strain in a mouse model of 5-Fluorouracil drug-induced mucositis. Administration of paraprobiotics and postbiotics reduced the neutrophil cells infiltrating into the small intestinal mucosa and ameliorated the intestinal epithelium architecture damaged by 5-FU. These ameliorative effects were associated with a downregulation of inflammatory markers (*Tlr2*, *Nfkb1*, *Il12*, *Il17a*, *Il1b*, *Tnf*), and upregulation of immunoregulatory *Il10* cytokine and the epithelial barrier markers *Ocln*, *Cldn1*, *2*, *5*, *Hp* and *Muc2*. Thus, heat-killed *L. delbrueckii* CIDCA 133 and supernatants derived from this strain were shown to be effective in reducing 5-FU-induced inflammatory damage, demonstrating them to be an alternative approach to the problems arising from the use of live beneficial microorganisms in clinical practice.

## 1. Introduction

Mucositis is characterized as a gastrointestinal (GIT) mucosal inflammation that frequently occurs in patients with cancer disease submitted to radiotherapy [1,2] and/or chemotherapy treatments (5-Fluorouracil, irinotecan, oxaliplatin, etc.) [3,4]. This debilitating condition is associated with pain and difficulty eating, leading to malnutrition, increasing the infection risk, alteration in the patient’s clinical status, and therapy delays [5,6,7].

Many strategies have been investigated to prevent and/or treat mucositis [5,8]. According to the pathobiology phases described by Sonis [3], the chemotherapy-induced dysbiotic intestinal microbiota has an essential role in the progression and severity of mucositis [9]. In this context, some probiotic microorganisms (e.g., *Lactobacillus* sp., *Bifidobacterium* sp., *Saccharomyces* sp.) have been intentionally explored as a therapeutic tool against the intestinal mucositis caused by chemotherapy treatment due to their ability to regulate the dysbiotic microbiota and their well-known anti-inflammatory properties, which mainly occur through the inhibition of the nuclear factor kappa B (NF-κB) signaling pathway with subsequent cellular and humoral immunomodulation [10,11,12,13]. Other positive effects attributed to some probiotic strains include the production of beneficial secreted metabolites such as short-chain fatty acids (SCFA) (e.g., acetate, propionate, and butyrate), inhibition of pathogenic bacterial growth by antimicrobial compounds (e.g., acetic and lactic acids and bacteriocins), and reinforcement of the intestinal barrier by mucus production and induced expression of tight junction proteins [14,15]. Probiotics can use these mechanisms in a strain-specific manner to protect and improve the intestinal mucosa healing process altered by chemotherapy.

Although some preclinical studies report the host’s beneficial effects related to “live” probiotics consumption [16,17,18,19,20], there are many questions regarding their safety concerns in clinical practice, especially in premature infants or immune-compromised patients. In these conditions, probiotics can translocate into the blood system and increase the risk of systemic infections [21,22,23,24]. The use of non-viable/inactivated probiotics, known as paraprobiotics (e.g., heat-killed probiotics) or products/molecules secreted or derived from these microorganisms, known as postbiotics (e.g., cell-free supernatant, surface layer proteins, cell lysates), besides minimizing or avoiding these possible adverse effects, has also been evaluated in anti-inflammatory therapies [25,26]. Paraprobiotics or postbiotics are an effective therapeutic alternative to gastrointestinal inflammation since they can also stimulate the host’s immune system to form an anti-inflammatory profile [15,27,28]. However, like probiotics, these biotics’ safety evaluations must also be performed before use for therapeutic purposes [25].

The anti-inflammatory properties and the intestinal barrier function conferred by the potential probiotic bacterium *Lactobacillus delbrueckii* subsp. *lactis* CIDCA 133 in a murine model of 5-FU-induced mucositis was previously demonstrated by our research group [29]. Ameliorative effects promoted by this strain were mainly associated with the reduction of inflammatory cells, improvement in mucus-producing goblet cell count, secretory IgA levels and intestinal permeability reduction, modulation of markers involved in NF-κB signaling pathway activation (*Tlr2*, *Nfkb1*, *Il6*, and *Il1b*), and regulation of tight junction protein expression [29,30]. Additionally, a probiogenomics study of this strain showed that its anti-inflammatory profile could be related to genes encoding secreted and membrane surface proteins able to interact with immune proteins involved in the NF-κB signaling pathway [31]. Regarding its safety concerns, it was reported that CIDCA 133 (10^7^ CFU/mL) did not cause blood hemolysis, mucin degradation, or epithelial damage in healthy mice, evidencing that this strain presents safety levels for probiotic application [32]. 

Few studies have evaluated the effects of heat-inactivated or cell-free supernatant on intestinal mucositis, mainly studies focused on the *Lactobacillus delbrueckii* species. The *L. delbrueckii* CIDCA 133 strain is a potential health-promoting bacterium. However, no beneficial effects have been reported with either the inactivation or extractable/secreted bioactive products derived from this strain. Thus, this work evaluated the anti-inflammatory properties of heat-killed and cell-free supernatants of *L. delbrueckii* CIDCA 133 in a mouse inflammation model induced by 5-Fluorouracil. 

## 2. Materials and Methods

### 2.1. Bacterial Culture Conditions

The strain *Lactobacillus delbrueckii* subsp. *lactis* CIDCA 133 (culture collection of Center for Research and Development in Food Cryotechnology of the National University of La Plata, Argentina) was grown on de Man, Rogosa and Sharpe (MRS) broth (Kasvi, São José dos Pinhais, Brazil) at 37 °C for 16 h. The strain was deposited at the Bacteria Collection from Environment and Health (CBAS) of the Oswaldo Cruz Foundation (FIOCRUZ) (access number: CBAS 815).

### 2.2. Lactobacillus delbrueckii CIDCA 133 Preparation (Viable, Heat-Inactivated, and Cell-Free Supernatant)

Viable bacteria were centrifuged (1520× *g* for 15 min at 4 °C), washed two times with sterile phosphate-buffered saline (PBS) 0.1 M (pH 7.2), and adjusted to a concentration of 10^9^ CFU/mL. The cell-free supernatant (CFS) was collected and buffered to a pH of 7.2 using TRIS 1 M (Sigma-Aldrich, St. Louis, MO, USA), as previously described by Prisciandaro et al. [33]. The CFS (15 mL) was sterilized with a 0.22 μm filter (Kasvi, São José dos Pinhais, Brazil), concentrated with Vivaspin 20 (weight cut-off 10,000 Kilodalton (kDa) (Sartorius, Gottingen, Germany), and stored at −80 °C until administration in mice. The heat-killed CIDCA 133 (10^9^ CFU/mL) was prepared by heat treatment (15 min, 121 °C). The inactivation process and CFS sterilization were confirmed after plating 100 μL of these biotics in MRS agar (Kasvi, São José dos Pinhais, Brazil). 

### 2.3. Animals

Six-week-old conventional BALB/c mice (males) weighing 20–24 g were obtained from the Bioterism Center (CEBIO) at the Federal University of Minas Gerais (UFMG, Belo Horizonte, Brazil). This study was approved by the Local Animal Experimentation Ethics Committee (CEUA-UFMG, Protocol n◦ 112/2020). Mice were housed in ventilated polycarbonate cages under controlled room conditions: 12 h light/dark cycle, temperature 25 ± 2 °C, and ad libitum access to standard chow and water. All procedures were conducted according to Brazilian Association for Laboratory Animal Science (SBCAL) guidelines.

### 2.4. Experimental Design

**Figure 1:**
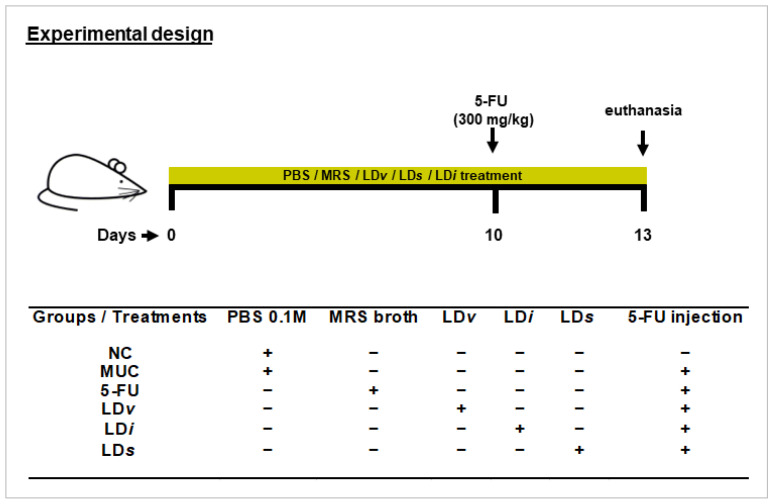
Experimental design protocol. Mice received gavage with 300 μL of PBS 0.1 M (NC and MUC group), MRS broth (5-FU group), viable CIDCA 133 (LD*v* group), heat-inactivated CIDCA 133 (LD*i* group), or cell-free supernatant (LD*s*) for 13 days. Mice were inflamed with a single dose of 5-Fluorouracil (300 mg/kg) on the 10th day. Symbols indicate (+) presence or (−) absence of treatment.

Mice were split into six experimental groups (*n* = six animals per group): negative control (NC), inflamed (MUC), inflamed (5-FU), inflamed and treated with viable CIDCA 133 (LD*v*), heat-killed inactivated CIDCA 133 (LD*i*), and CIDCA 133 cell-free supernatant (LD*s*). Mice received doses of 300 μL containing 10^9^ CFU/mL of CIDCA 133 (LD*v* and LD*i* groups) or 300 μL of CIDCA 133 supernatants (LD*s* group) for 13 days by intragastric gavage. Control groups received 300 μL of PBS 0.1M solution (NC and MUC groups) or MRS broth (5-FU group) by the same route. On the 10^th^ day, mice (MUC, 5-FU, LD*v*, LD*i*, and LD*s* groups) were inflamed intraperitoneally with a single injection of 5-Fluorouracil (Fauldfluor^®^) (300 mg/kg) (Libbs, São Paulo, Brazil) [29]. Sterile saline solution injection (NaCl 0.9%) (Vetec, Rio de Janeiro, Brazil) (Figure 1) was administered to the negative control group. At the end of the experimental procedure, 72 h after mucositis induction, mice were euthanized by an anesthetic overdose (ketamine 300 mg/kg and xylazine 30 mg/kg solution) (Syntec, Tamboré, Brazil), and afterward, ileum sections were collected for analysis. 

### 2.5. Inflammatory Cell Infiltration

The recruitment of neutrophils and eosinophils to intestinal mucosa was performed by myeloperoxidase (MPO) [34] and eosinophil peroxidase (EPO) [35] enzymatic activity assays, respectively. For MPO, ileum tissues (100 mg) were homogenized with 1.9 mL of buffered solution (pH 4.7) (NaCl 0.1 M (LabSynth, Diadema, Brazil), NaH_2_PO_4_ 0.02 M (LabSynth, Diadema, Brazil), and Na_2_EDTA 0.015 M (LabSynth, Diadema, Brazil)) and centrifuged (9500× *g* for 10 min at 4 °C). After a hypotonic process (0.2% NaCl solution plus 1.6% NaCl solution containing glucose 5% (LabSynth, Diadema, Brazil)) and centrifugation (9500× *g* for 10 min at 4 °C), the pellet was homogenized in a NaH_2_PO_4_ (0.05 M) solution (pH 5.4) containing 0.5% hexadecyltrimethylammonium bromide (Sigma-Aldrich, St. Louis, MO, USA) and submitted to lysis process with three cycles of freeze-thaw in liquid nitrogen. After the second centrifugation (9500× *g* for 15 min at 4 °C), the supernatant was collected for colorimetric assay. Thus, 25 μL of supernatant was added to 25 μL of 3,3,5,5′-Tetramethylbenzidine 1.6 mM (Sigma-Aldrich, St. Louis, MO, USA) previously diluted in dimethyl sulfoxide (Sigma-Aldrich, St. Louis, MO, USA). After adding 100 μL of H_2_O_2_ 0.5 mM (Vetec, Rio de Janeiro, Brazil), the plates were incubated at 37 °C for 5 min. 

For EPO, 100 mg of ileum tissues were homogenized in 1.9 mL of PBS 0.1 M (pH 7.4). After centrifugation (9500× *g* for 15 min at 4 °C), a hypotonic process (0.2% NaCl solution, plus 1.6% NaCl solution containing glucose 5%) and second centrifugation (9500× *g* for 15 min at 4 °C), the pellet was homogenized in a PBS 0.1 M solution (pH 7.4) containing 0.5% hexadecyltrimethylammonium bromide and submitted to lysis process with three cycles of freeze-thaw in liquid nitrogen. After another centrifugation (9500× *g* for 15 min at 4 °C), the supernatant was collected for colorimetric assay. For this purpose, 75 μL of supernatant were added to 75 μL of O-phenylenediamine 1.5 mM (Sigma-Aldrich, St. Louis, MO, USA), diluted in Tris–HCl 0.075 mM (pH 8) plus H_2_O_2_ 6.6 mM, and incubated at 20 °C for 30 min. Both enzymes’ reactions were stopped by adding 50 μL of H_2_SO_4_ 1 M (Vetec, Rio de Janeiro, Brazil). The absorbance of the products of the enzymatic assays was measured at 450 nm (MPO) and 492 nm (EPO) on a microplate spectrophotometer (Bio-Rad 450 model, Bio-Rad Laboratories). The results were expressed as MPO or EPO arbitrary units/mg of tissue based on absorbance. 

### 2.6. Gene Expression of Cytokines and Epithelial Barrier Markers

#### 2.6.1. Total RNA Isolation

The profile of cytokines and genetic markers involved with epithelial barrier function was evaluated through gene expression. For this, ileum total RNA isolation was carried out using Pure Link™ RNA Mini Kit (Invitrogen, Carlsbad, CA, USA), according to the recommended protocol. Samples were analyzed using the NanoDrop 2000 spectrophotometer (Thermo Scientific, Waltham, MA, USA) and 1.5 % agarose gel to verify the total RNA concentration and quality. Residual genomic DNA was removed using the Turbo DNA-free™ Kit (Invitrogen, Carlsbad, CA, USA). Complementary DNA was produced with 2 μg of RNA using the High-Capacity cDNA Reverse Transcription kit (Applied Biosystems™, ThermoFisherC, Waltham, MA, USA), according to the recommended protocol.

#### 2.6.2. Quantitative PCR (qPCR)

Quantitative PCR was carried out with the PowerUp™ SYBR^®^ Green Master Mix (ThermoFisher) on the ABI PRISM 7900HT Sequence Detection System (Applied Biosystems™) under the following steps: 95 °C for 10 min, and 40 cycles of 95 °C for 15 s, and 60 °C for 1 min. The Toll-like receptor 2 (*Tlr2*), nuclear factor-kappa B p105 subunit (*Nfkb1*), interleukins b (*Il1b*), 10 (*Il10*), 12 (*Il12*), 17-alfa (*Il17a*), tumor necrosis factor (*Tnf*), transforming growth factor beta-1 (*Tgfb1*), mucin 2 (*Muc2*), claudin 1 (*Cldn1*), 2 (*Cldn2*), and 5 (*Cldn5*), zonulin (*Hp*), and occludin (*Ocln*) were used as gene-specific primers (Table 1) [36,37,38,39,40]. Gene expression results were analyzed following the 2^−ΔΔCT^ method using GAPDH (*Gapdh*) and β-actin (*Actb*) as endogenous references. 

### 2.7. Histological and Morphometric Analysis

The ileum sections were collected for histological analysis, washed with PBS 0.1 M, longitudinally opened, rolled up, and immersed in 10% buffered formaldehyde solution (Neon, Suzano, Brazil) for 24 h until tissue fixation. This material was embedded in paraffin, and sections of 4 μm thickness were stained with hematoxylin and eosin (HE). The histological inflammation score was examined by a pathologist and evaluated according to the Soares et al. [41] method. Histological images were captured by a BX41 optical microscope (Olympus, Tokyo, Japan) with a 20× magnification objective, and the morphometric parameters were evaluated by measuring 20 villi height and 20 crypt depth with the ImageJ 1.51j.8 software (National Institutes of Health, Bethesda, MD, USA).

### 2.8. Statistical Analysis

The results are presented as the mean ± standard deviation. Data were evaluated by one-way ANOVA followed by Tukey’s post hoc test (parametric data) or by the Kruskal–Wallis test and post-tested by Dunn’s test (nonparametric data). Graphs were generated and data analysis performed using GraphPad Prism 8.0 software (GraphPad Software) and a *p*-value < 0.05.

## 3. Results

### 3.1. Heat-Killed Lactobacillus delbrueckii CIDCA 133 Improved Weight Loss in Chemotherapy-Inflamed Mice

**Figure 2:**
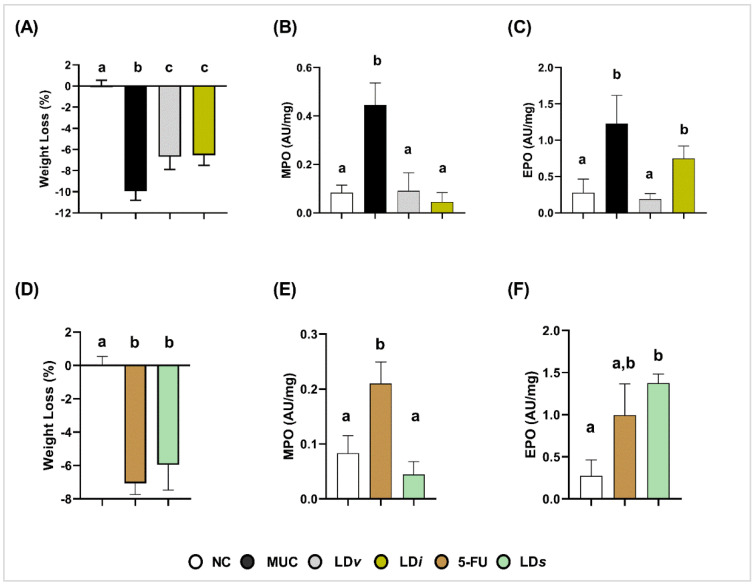
Protective effect of heat-killed (LD*i*) and cell-free supernatant (LD*s*) of *L. delbrueckii* CIDCA 133 on 5-FU-induced body weight loss and recruitment of polymorphonuclear cells. (**A**–**C**) Viable and heat-killed CIDCA 133. (**D**–**F**) CIDCA 133 supernatant. Different letters (a, b, c) indicate statistically significant differences (*p* < 0.05) by ANOVA followed by Tukey’s (**A**–**E**) or by Kruskal–Wallis test followed by Dunn’s post hoc test (**F**).

Body weight loss was higher in the MUC (−9.9 ± 0.85%) (Figure 2A) and 5-FU (−7.0 ± 0.68%) (Figure 2D) groups when compared to the NC group (0.00 ± 0.5%) (*p* < 0.0001) (Figure 2A,D). Treatment with viable (LD*v*) (−6.6 ± 1.1%) and heat-killed CIDCA 133 (LD*i*) (−6.5 ± 0.97%) (Figure 2A) improved the weight loss (*p* < 0.001). However, the cell-free supernatant (LD*s*) (−5.95 ± 1.51%) had no protective effect on this parameter (*p* = 0.26) (Figure 2D).

### 3.2. Heat-Killed and Cell-Free Supernatant of Lactobacillus delbrueckii CIDCA 133 Reduced Levels of Myeloperoxidase Activity

Inflammatory infiltrates of neutrophils and eosinophils in ileum mucosa were assessed by levels of their respective myeloperoxidase (MPO) and eosinophil peroxidase (EPO) enzymatic activities. High levels of MPO (MUC: 0.44 ± 0.09 AU/mg, Figure 2B; 5-FU: 0.21 ± 0.03 AU/mg, Figure 2E) and EPO (MUC: 1.23 ± 0.38 AU/mg, Figure 2C; 5-FU: 0.99 ± 0.37 AU/mg, Figure 2F) enzymatic activities were detected in the ileum mucosa of inflamed mice when compared to the control group (MPO: 0.08± 0.03 AU/mg; EPO: 0.27 ± 0.19 AU/mg) (Figure 2B,C,E) (*p* < 0.0001). Treatment with viable CIDCA 133 (LD*v*) reduced the level activity of both enzymes (MPO: 0.09± 0.07 AU/mg, *p* < 0.0001; EPO: 0.18 ± 0.07 AU/mg, *p* < 0.0001) (Figure 2B,C). Positive effects of heat-killed (LD*i*) (0.09 ± 0.07 AU/mg, *p* < 0.0001) and cell-free supernatant (LD*s*) (0.04 ± 0.02 AU/mg, *p* < 0.0001) were only observed against MPO enzyme levels (Figure 2B,E). No protective effect of heat-killed (LD*i*) (0.74 ± 0.17 AU/mg, *p* = 0.06) (Figure 2C) and cell-free supernatant of CIDCA 133 (LD*s*) (1.37 ± 0.10 AU/mg, *p* = 0.7130) (Figure 2F) was observed with respect to EPO levels.

### 3.3. Heat-Killed and Cell-Free Supernatant of Lactobacillus delbrueckii CIDCA 133 Modulated the Gene Expression of Inflammatory Cytokines

**Figure 3:**
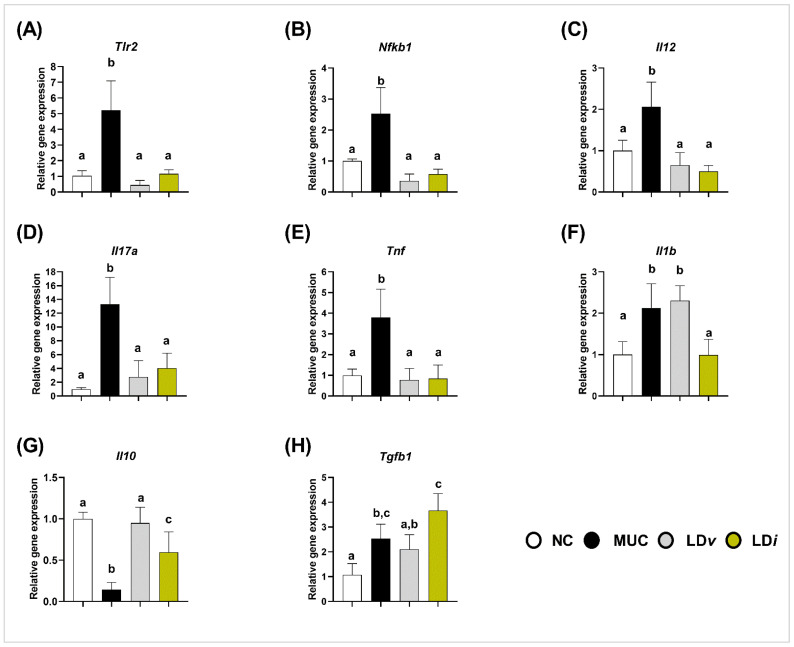
Heat-killed (LD*i*) *L. delbrueckii* CIDCA 133 modulated gene expression of inflammatory cytokines. (**A**) *Tlr2*, (**B**) *Nfkb1*, (**C**) *Il12*, (**D**) *Il17a*, (**E**) *Tnf,* (**F**) *Il1b*, (**G**) *Il10* and (**H**) *Tgfb1*. Different letters (a, b, c) indicate statistically significant differences (*p* < 0.05) by ANOVA followed by Tukey’s post hoc test.

The gene expression of pro-inflammatory (*Il1b*, *Il12*, *Il17a*, and *Tnf*) and anti-inflammatory (*Il10* and *Tgfb1*) cytokines was performed to evaluate the immunoregulatory effects of heat-killed and cell-free supernatant of *L. delbrueckii* CIDCA 133. Compared to the control group, mice in the MUC group presented an upregulation in the gene expression of *Tlr2* (*p* < 0.001) (Figure 3A), *Nfkb1* (*p* < 0.001) (Figure 3B), *Il12* (*p* < 0.01) (Figure 3C), *Il17a* (*p* < 0.001) (Figure 3D), *Tnf* (*p* < 0.01) (Figure 3E), *Il1b* (*p* < 0.01) (Figure 3F), and *Tgfb1* (*p* < 0.05) (Figure 3H), and a downregulation of the immunoregulatory cytokine *Il10* (*p* < 0.0001) (Figure 3G). 

Modulation of these inflammatory markers was observed after treatment with CIDCA 133. Consumption of viable bacteria (LD*v*) reduced the mRNA expression of *Tlr2* (*p* < 0.0001) (Figure 3A), *Nfkb1* (*p* < 0.0001) (Figure 3B), *Il12* (*p* < 0.001) (Figure 3C), *Il17a* (*p* < 0.001) (Figure 3D), *Tnf* (*p* < 0.0001) (Figure 3E), and increased *Il10* (*p* < 0.0001) (Figure 3G) when compared to the MUC group. Heat-killed (LD*i*) treatment downregulated *Tlr2* (*p* < 0.0001) (Figure 3A), *Nfkb1* (*p* < 0.0001) (Figure 3B), *Il12* (*p* < 0.0001) (Figure 3C), *Il17a* (*p* < 0.01) (Figure 3D), *Tnf* (*p* < 0.0001) (Figure 3E), and *Il1b* (*p* < 0.01) (Figure 3F), and upregulated the gene expression of *Il10* (*p* < 0.01) (Figure 3G). No differences were observed between the MUC and LD*i* groups in *Tgfb1* expression (*p* = 0.112) (Figure 3H).

**Figure 4:**
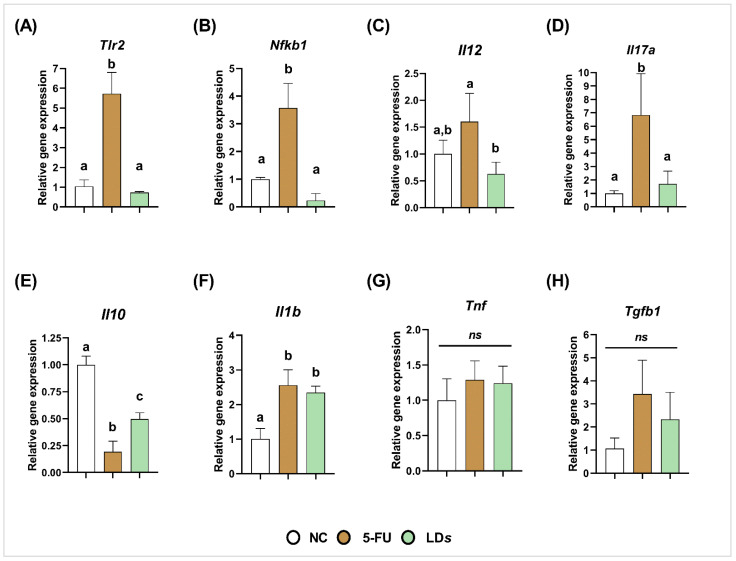
Cell-free supernatant ((LD*s*) of *L. delbrueckii* CIDCA 133 downregulated 5-FU-induced gene expression of pro-inflammatory cytokines. (**A**) *Tlr2*, (**B**) *Nfkb1*, (**C**) *Il12*, (**D**) *Il17a*, (**E**) *Il10,* (**F**) *Il1b*, (**G**) *Tnf* and (**H**) *Tgfb1*. Different letters (a, b, c) indicate statistically significant differences (*p* < 0.05) by ANOVA followed by Tukey’s posttest. ns indicates no statistically significant differences.

On the other hand, the 5-FU group exhibited upregulation of *Tlr2* (*p* < 0.0001) (Figure 4A), *Nfkb1* (*p* < 0.001) (Figure 4B), *Il17a* (*p* < 0.05) (Figure 4D) and *Il1b* (*p* < 0.0001) (Figure 4F), and downregulation of *Il10* (*p* < 0.01) (Figure 4E). Treatment with the cell-free supernatant of CIDCA 133 (LD*s*) downregulated the gene expression of *Tlr2* (*p* < 0.0001) (Figure 4A), *Nfkb1* (*p* < 0.0001) (Figure 4B), pro-inflammatory cytokines *Il12* (*p* < 0.01) (Figure 4C), and *Il17a* (*p* < 0.05) (Figure 4D), and upregulated anti-inflammatory cytokine *Il10* (*p* < 0.01) (Figure 4E) when compared to the 5-FU group. No differences were observed in *Il1b*, *Tnf*, and *Tgfb1* gene expression between 5-FU and LD*s* groups (*p* > 0.05) (Figure 4F–H).

### 3.4. Heat-Killed and Cell-Free Supernatant of Lactobacillus delbrueckii CIDCA 133 Regulated Genes Related to Intestinal Epithelial Barrier 

**Figure 5:**
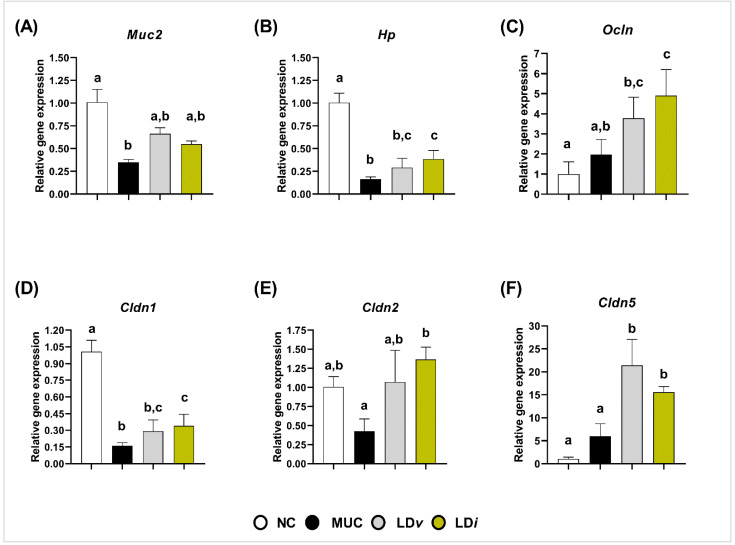
Heat-killed (LD*i*) *L. delbrueckii* CIDCA 133 modulated mRNA expression of markers involved in epithelial barrier function. (**A**) mucin 2, (**B**) zonulin, (**C**) occludin (**D**) claudin 1, (**E**) claudin 2 (**F**) claudin 5. Different letters (a, b, c) indicate statistically significant differences (*p* < 0.05) by ANOVA followed by Tukey’s posttest (**B**–**D**,**F**) or by Kruskal–Wallis test followed by Dunn’s post hoc test (**A**,**E**).

The regulatory effect of heat-killed and cell-free supernatant of *L. delbrueckii* CIDCA 133 on epithelial barrier protection was evaluated through the gene expression of mucin 2 and tight junction proteins (*Cldn1*, *Cldn2*, *Cldn5*, *Hp* and *Ocln*). The MUC group exhibited a downregulation in the mRNA expression of *Muc2* (*p* < 0.01) (Figure 5A), *Hp* (*p* < 0.0001) (Figure 5B), and *Cldn1* (*p* < 0.0001) (Figure 5D) when compared to the control group. The treatment with viable *L. delbrueckii* CIDCA 133 (LD*v*) only upregulated the *Cldn5* (*p* < 0.0001) (Figure 5F) gene expression; however, upregulation of *Hp* (*p* < 0.05) (Figure 5B), *Ocln* (*p* < 0.01) (Figure 5C), *Cldn1* (*p* < 0.05) (Figure 5D), *Cldn2* (*p* < 0.01) (Figure 5E), and *Cldn5* (*p* < 0.01) (Figure 5F) was observed after heat-killed CIDCA 133 treatment (LD*i*). No differences were observed in the mRNA expression of *Muc2* (Figure 5A) after heat-killed CIDCA 133 treatment and in *Hp* (Figure 5B), *Ocln* (Figure 5C), *Cldn1* (Figure 5D), and *Cldn2* (Figure 5E) after viable CIDCA 133 (LD*v*) treatment (*p* > 0.05).

**Figure 6:**
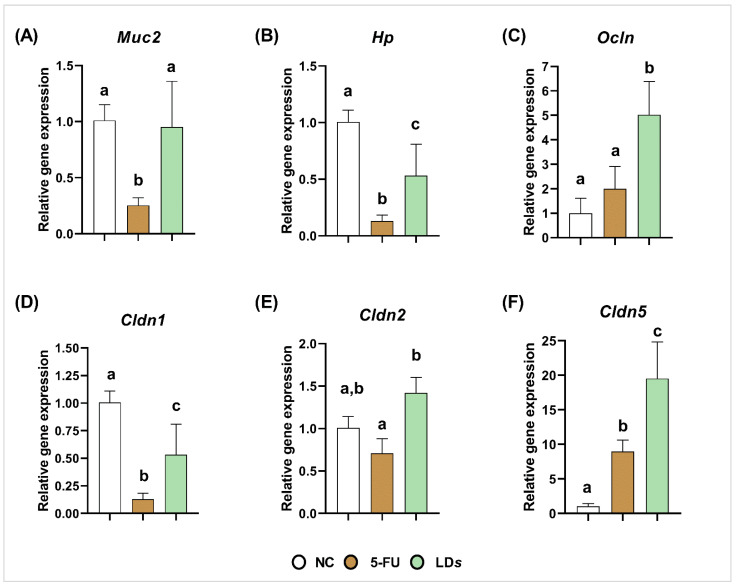
Cell-free supernatant (LD*s*) of *L. delbrueckii* CIDCA 133 modulated mRNA expression of markers involved in epithelial barrier function. (**A**) mucin 2, (**B**) zonulin, (**C**) occludin (**D**) claudin 1, (**E**) claudin 2 (**F**) claudin 5. Different letters (a, b, c) indicate statistically significant differences (*p* < 0.05) by ANOVA followed by Tukey’s posttest (**A**–**D**,**F**) or by Kruskal–Wallis test followed by Dunn’s post hoc test (**E**).

The 5-FU group also exhibited a downregulation in the mRNA expression of *Muc2* (*p* < 0.01) (Figure 6A), *Hp* (*p* < 0.0001) (Figure 6B), and *Cldn1* (*p* < 0.0001) (Figure 6D), but upregulated expression of *Cldn5* (*p* < 0.05) (Figure 6F) when compared to the control group. However, after treatment with cell-free supernatant of CIDCA 133 (LD*s*), an upregulation of mRNA expression of *Muc2* (*p* < 0.01) (Figure 6A), *Hp* (*p* < 0.05) (Figure 6B), *Ocln* (*p* < 0.01) (Figure 6C), *Cldn1* (*p* < 0.05) (Figure 6D), *Cldn2* (*p* < 0.01) (Figure 6E), and *Cldn5* (*p* < 0.01) (Figure 6F) was observed. 

### 3.5. Heat-Killed and Cell-Free Supernatant of Lactobacillus delbrueckii CIDCA 133 Improved Epithelium Intestinal Architecture 

**Figure 7:**
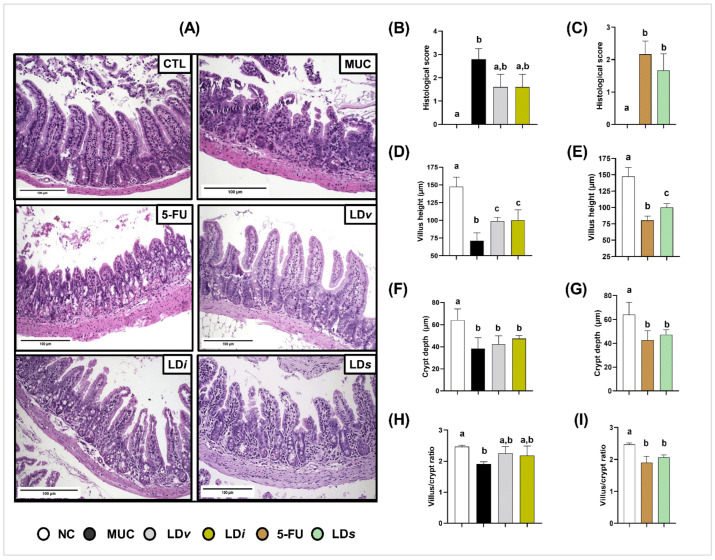
Heat-killed (LD*i*) and cell-free supernatant (LD*s*) of *L. delbrueckii* CIDCA 133 ameliorated intestinal architecture damage induced by 5-FU administration. (**A**) Ileum mucosa histopathology images (objective: ×20, scale 100 µm); (**B**,**F**) histopathological score; (**C**,**G**) villus height; (**D**,**H**) crypt depth; and (**E**,**I**) villus height to crypt depth ratio, respectively. Different letters (a, b, c) indicate statistically significant differences (*p* < 0.05) by Kruskal–Wallis test followed by Dunn’s post hoc test (**B**,**F**) or ANOVA followed by Tukey’s post hoc test (**C**–**E**,**G**–**I**).

Histopathological analysis demonstrated that mice inflamed with 5-Fluorouracil chemotherapy presented intestinal architecture alterations characterized by intense inflammatory cell infiltration into the lamina propria and villi, degeneration of goblet cells, villus shortening, and crypt necrosis when compared to the control group (Figure 7A), corroborating the histopathological score (Figure 7B,C) and morphometric analysis (Figure 7D–I). 

Treatment with viable (LD*v*), heat-killed (LD*i*) (Figure 7D), and cell-free supernatant (LD*s*) (Figure 7E) of CIDCA 133 had a protective effect on villus shortening and inflammatory cell infiltration into the lamina propria and villus, improving the epithelium architecture of the ileum section (Figure 7A). After consumption of these biotic agents, no beneficial effects were observed in the mice´s histopathological score, crypt depth, or villus/crypt ratio (Figure 7B,C,F–I).

## 4. Discussion

The anti-inflammatory properties of viable *L. delbrueckii* CIDCA 133 have been previously reported [29,30,31]. This study evaluated the anti-inflammatory properties of the bacterial inactivation or secreted bioactive products derived from this strain in a 5-Fluorouracil drug-induced mouse intestinal inflammation model. 

The mucosal destruction by chemotherapy leads to lower absorption of nutrients, influencing body weight loss [3,41,42]. Our results showed no protective effect against weight loss with the cell-free supernatant of *L. delbrueckii* CIDCA 133. This lack of protection was possibly due to the removal of small molecules derived from CIDCA 133 after supernatant concentration with Vivaspin 20, thus reducing their interaction with intestinal epithelial cells to provide beneficial properties to the host. These data are similar to the findings of Prisciandaro et al. [33], demonstrating that *Escherichia coli* Nissle 1917 and *Limosilactobacillus fermentum* BR11 supernatant did not improve weight loss in mice with intestinal mucositis [33]. On the other hand, the consumption of heat-killed *L. delbrueckii* CIDCA 133 ameliorated the weight loss in BALB/c mice after 5-FU administration, similarly to viable bacteria, as previously reported by De Jesus et al. [29], and also corroborating with Trindade et al. [43], who reported that heat-killed *Lacticaseibacillus rhamnosus* CGMCC1.3724 strain influenced weight recovery of 5-FU inflamed mice. 

The intestinal architecture alteration resulting from the inflammatory process is one of the most critical features in the pathobiology of chemotherapy-induced mucositis [44,45]. Our results showed that the heat-killed and cell-free supernatants of *L. delbrueckii* CIDCA 133 improved the ileum epithelium architecture destroyed by 5-FU administration. This protective property can be linked to the ability of these biotics to attenuate villus shortening, upregulate the immunoregulatory cytokine *Il10* at the mRNA level, and reduce the inflammatory markers (e.g., *Tlr2*, *Nfkb1*, pro-inflammatory cytokines *Il1b*, *Il12*, *Il17a*, *Tnf*, and neutrophil infiltrates). These findings are supported by other studies in which administration either of inactivated *Lacticaseibacillus rhamnosus* CGMCC1.3724 [43], *Lactiplantibacillus plantarum* Zhang-LL [28], and VSL#3 therapy composed of *Bifidobacterium breve*, *B. longum*, *B. infantis*, *L. plantarum*, *Lb. bulgaricus*, *L. casei*, *L. acidophilus*, and *Streptococcus salivarius* [46], or secreted products derived from beneficial microorganisms, such as supernatant of mulberry leaf extract fermented by *L. acidophilus* A4 [47] and supernatant of *Faecalibacterium prausnitzii* [27], ameliorated the intestinal inflammatory process through modulation of inflammatory markers, such as myeloperoxidase activity levels, Tregs CD4^+^ Foxp3^+^ cells, pro-inflammatory cytokines IL1β, IFNγ, IL17A, TNFα, and anti-inflammatory IL10 and IL4 cytokines, thus restoring the intestinal homeostasis. 

Interestingly, our results show that the transcripts levels of immunoregulatory cytokine TGFβ1 were upregulated after 5-FU administration and maintained after viable and heat-inactivated CIDCA 133 administration. Furthermore, we only observed a reduction in eosinophil inflammatory infiltrate with viable CIDCA 133 treatment. The protective effect on this parameter was not observed with the paraprobiotic and postbiotic forms of CIDCA 133.

TGFβ1 is an abundant cytokine in the intestinal mucosa with pleiotropic effects. The anti-inflammatory or pro-inflammatory property of this immunoregulatory factor occurs through cellular and environmentally dependent contexts [48,49]. Increased TGFβ levels were observed in the 5-FU-induced inflammation model [50,51,52]. On the other hand, IL10 cytokine levels were reduced [30,53,54,55]. Evidence shows that in the absence/reduction of IL10 cytokine in the intestinal inflammation context, levels of TGFβ1 are increased [19,56]. Thus, we suggest that, due to the reduction of IL10 transcript levels, paraprobiotics and postbiotics of CIDCA 133 maintain the mRNA level of the TGFβ1 cytokine as a compensatory anti-inflammatory mechanism to control tissue damage and restore gut homeostasis disrupted by 5-Fluorouracil chemotherapy.

Regarding infiltrating eosinophils, these cells reside in the intestine, in both normal and inflammatory states [57]. Pieces of evidence show that eosinophil activation and migration can be regulated by microbiota metabolites (e.g., SCFA) [58]. Thus, we believed that SCFA production and microbiota interaction might be a mechanism used by CIDCA 133 in its beneficial effect, suggesting that its viability is necessary to reduce 5-FU-induced eosinophil infiltration in the ileum mucosa.

The anti-inflammatory profile of heat-inactivated and cell-free supernatant of *L. delbrueckii* CIDCA 133 was, at least in part, similar to that of its viable form. We believe that these results can be linked to extracellular molecules and cell surface-associated components (e.g., LtaS, SLAP, HtrA, MucBP, and PrtB) identified in the strain genome [31] since these genetic factors interacted the most with human immune protein receptors associated with NF-κB signaling pathway regulation, including NFKB1 protein. These molecular markers were also associated as possibly genetic factors of viable CIDCA 133 involved with its anti-inflammatory properties through the upregulation of immunoregulatory *Tgfb1* and *Il10* cytokines and downregulation of *Nfkb1* gene expression in vivo [30,31]. 

Cell surface-associated components or extracellular molecules (e.g., surface layer proteins, lipoteichoic acids, exopolysaccharides) can stimulate the immune system of the host through their interaction with intestinal epithelial cells via specific receptors such as Toll-like (TLRs) and NOD receptors (NLRs), thus regulating the balance between the anti-inflammatory and pro-inflammatory responses induced by different signaling pathways, such as NF-κB and MAPK [9,59]. Immunoregulation promoted by these bacterial factors has been previously reported by Chandhni et al. [60], who showed that the extractable surface proteins derived from *Lactiplantibacillus plantarum* MTCC 5690, *Lactobacillus acidophilus* NCFM and *Limosilactobacillus fermentum* MTCC 5689 ameliorated DSS or TNBS-induced acute intestinal inflammation by increasing immunoregulatory IL10 cytokine and decreasing leukocyte infiltration, TNFα, and IFNγ pro-inflammatory markers [60]. Similar findings were also reported by Deutsch et al. [61] when demonstrating that surface proteins, such as SlpB and SlpE, are the biological agents responsible for the anti-inflammatory properties of *Propionibacterium freudenreichii* strains. These proteins increased the production of the immunoregulatory cytokine IL10 and modulated pro-inflammatory cytokine TNFα, IFNγ, and IL6 responses of human PBMCs cells [61]. Anti-inflammatory properties of extracellular polysaccharides of *L. delbrueckii* TUA4408L were also reported. These bacterial components attenuated enterotoxigenic *Escherichia coli*-induced inflammatory response in porcine intestinal epitheliocytes by regulating TLR2/4 receptors and MAPK or NF-κB pathway activation and decreasing IL6, IL8, and monocyte chemoattractant protein-1 (MCP-1) levels [59]. 

It is also interesting to note that, despite the literature demonstrating an anti-inflammatory profile attributed to the probiotic-derived immune molecular effectors, there is evidence that some of these bioactive factors, such as the B7 peptides derived from the probiotic *Bifidobacterium longum*, can exacerbate the intestinal inflammatory process by increasing pro-inflammatory markers and immune cell activation (e.g., circulating antigen-presenting cells) [62]. Thus, these findings show that bioactive bacterial factors can elicit differential immune mechanisms depending on the context of the progression stage (early or late) of the inflammation [62]. In this context, despite surface-exposed components or secreted bioactive molecules appearing to be essential for leading the anti-inflammatory process in the host by CIDCA 133, further studies should be conducted to characterize the cytotoxic/pro-inflammatory profiles of these CIDCA 133-derived molecular components or metabolites, especially in severe inflammation models. 

The 5-FU chemotherapy-induced intestinal inflammation also leads to loss of epithelial barrier integrity via the depletion of tight junction proteins (TJs), reduction in goblet cell numbers, and increased intestinal permeability [37,45,53]. TJs proteins are part of an interconnected network of adhesion complexes that act as selective barriers between internal and external cellular environments, thus controlling the passage of pathogens and other foreign molecules [63,64]. The reduction in mRNA expression of mucin 2 and tight junction proteins zonulin, claudin 1, and claudin 2 observed in the inflamed mice was attenuated by both heat-killed and cell-free supernatants of *L. delbrueckii* CIDCA 133. These results are consistent with data obtained by De Jesus et al. [29] and Barroso et al. [30], who demonstrated that viable *L. delbrueckii* CIDCA 133 reduced intestinal permeability and goblet cell degeneration and upregulated the gene expression of tight junction proteins (*Cldn1, Fr11, Hp*) altered by 5-FU chemotherapy, thus being essential to reduce the translocation of toxins and pathogenic bacteria, and consequently the inflammation amplification process in the intestinal mucosa [45]. 

The improvement in levels of mucins and tight junction proteins after treatment with heat-inactivated beneficial microorganisms and their supernatants has been previously reported. For example, mulberry leaf extract supernatant fermented by *L. acidophilus* upregulated the gene expression of *Muc2* and *Muc5AC* in 5-FU-inflamed mice [47]. Furthermore, secreted factors of *E. coli* Nissle 1917 attenuated the epithelial barrier disruption induced by enteropathogenic *E. coli* in Caco-2 cells by enhancing the gene expression of tight junction proteins zonulin-1, claudin-14, and claudin-2 [65]. Trindade et al. [43] also reported that paraprobiotics *L. rhamnosus* CGMCC1.3724 reduced intestinal permeability induced by 5-FU and enhanced *Muc2* gene expression. Promising results were also reported with inactivated *B. longum* K2-21-4, which modulated the gene expression of claudin-1, zonulin-1, and occludin disrupted by lipopolysaccharide (LPS) in colon epithelial cells [66]. 

Claudins are the essential transmembrane proteins participating in complex strand networks that regulate the paracellular permeability and maintain the intestinal mucosal barrier function [63,67,68]. The discrepancies in these proteins’ expressions may be related to their different interactions with other membrane compartments [63]. Surprisingly, our results showed that 5-FU administration increased the gene expression of claudin-5, contrary to other studies [9,30,37,45,53,69,70]. One possible explanation for this outcome is that this protein was upregulated as a compensatory response due to decreased expression of other tight junction proteins caused by 5-FU. A similar outcome was observed by Li et al. [45], who demonstrated that 5-FU enhanced the expression levels of tight junction ZO-1 and adhesion molecules such as JAM-A while reducing colonic occludin levels. Another piece of evidence indicates that upregulation of claudin-5 can be a mechanism to accelerate intestinal epithelial differentiation to maintain epithelial homeostasis [71].

**Figure 8:**
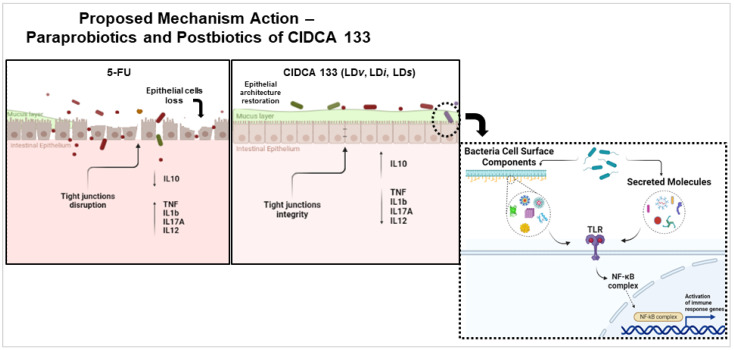
Proposed mechanism of action of paraprobiotics and postbiotics of *L. delbrueckii* CIDCA 133 in intestinal mucositis induced by 5-FU chemotherapy.

Based on our results and the pathobiology of mucositis proposed by Sonis [3], we believe that a proposed mechanism used for paraprobiotics CIDCA 133 and its postbiotics to reduce intestinal mucosal inflammation and reinforce the epithelial barrier occurs due to surface-associated components or secreted molecules able to interact with intestinal epithelial cells via specific pattern recognition receptors (PRRs), mainly TLR receptors. This process would control the dysbiotic intestinal microbiota and stimulate goblet cells to secrete mucin, reinforcing the epithelial barrier. On the other hand, the activation of these epithelial cell receptors by these biotics would induce intestinal immune cells to produce the immunoregulatory IL10 cytokine to control activation of the NF-κB signaling pathway and, consequently, regulate the balance between anti-inflammatory and pro-inflammatory immune responses. This process would reduce the migration of inflammatory cells to the mucosa, with subsequent reduction of oxidative stress generated by these cells. All the above processes would decrease tissue damage, reduce the disruption of the tight junction proteins, improve villus length due to migration of enterocytes produced by stem cells in crypts, and, thus, contribute to intestinal architecture recovery and homeostasis (Figure 8). However, we emphasize that more studies should be carried out to better elucidate the immunological, molecular, and cellular mechanisms of action of paraprobiotics and postbiotics from CIDCA 133 in intestinal mucositis and other inflammation models, including knock-out gene mouse models, proteomics, SCFA analysis, microbiota regulation, evaluation of cytokine levels, and activation of immune regulatory cells.

## 5. Conclusions

Altogether, our results demonstrated that heat-killed and cell-free supernatants of the *Lactobacillus delbrueckii* CIDCA 133 strain protected the intestinal mucosa from epithelial damage caused by the 5-FU drug. These ameliorative effects were detectable morphologically and had a profile similar to that of the viable form of CIDCA 133. Modulation of inflammatory parameters and epithelial barrier dysfunction was the primary mechanism used by these biotics to protect the intestinal mucosa from epithelial destruction caused by the 5-FU drug, suggesting them to be an attractive alternative therapeutic approach against intestinal damage induced by chemotherapy and to the problems arising from the use of live beneficial microorganisms in clinical practice.

## Figures and Tables

**Table 1:** qPCR primer sequences.

Gene	Primer Forward	Primer Reverse	Reference
*Gapdh*	TCACCACCATGGAGAAGGC	GCTAAGCAGTTGGTGGTGCA	[36]
*Actb*	GCTGAGAGGGAAATCGTGCGTG	CCAGGGAGGAAGAGGATGCGG	[38]
*Tlr2*	ACAATAGAGGGAGACGCCTTT	AGTGTCTGGTAAGGATTTCCCAT	[39]
*Nfkb1*	GTGGAGGCATGTTCGGTAGTG	TCTTGGCACAATCTTTAGGGC	[40]
*Il12p40*	GGAAGCACGGCAGCAGAATA	AACTTGAGGGAGAAGTAGGAATGG	[36]
*Il17a*	GCTCCAGAAGGCCCTCAGA	AGCTTTCCCTCCGCATTGA	[36]
*Tgfb1*	TGACGTCACTGGAGTTGTACGG	GGTTCATGTCATGGATGGTGC	[36]
*Il10*	GGTTGCCAAGCCTTATCGGA	ACCTGCTCCACTGCCTTGCT	[36]
*Tnf*	ACGTGGAACTGGCAGAAGAG	CTCCTCCACTTGGTGGTTTG	[37]
*Il1b*	CTCCATGAGCTTTGTACAAGG	TGCTGATGTACCAGTTGGGG	[37]
*Muc2*	GATGGCACCTACCTCGTTT	GTCCTGGCACTTGTTGGAAT	[38]
*Cldn1*	TCCTTGCTGAATCTGAACA	AGCCATCCACATCTTCTG	[38]
*Cldn2*	GTCATCGCCCATCAGAAGAT	ACTGTTGGACAGGGAACCAG	[38]
*Cldn5*	GCTCTCAGAGTCCGTTGACC	CTGCCCTTTCAGGTTAGCAG	[38]
*Ocln*	ACTCCTCCAATGGACAAGTG	CCCCACCTGTCGTGTAGTCT	[38]
*Hp*	CCACCTCTGTCCAGCTCTTC	CACCGGAGTGATGGTTTTCT	[38]

## Data Availability

Data are available by contacting the corresponding author.
